# Host and Surgical Factors Affecting the Frequency and Duration of Revision Endoscopic Sinus Surgery

**DOI:** 10.7759/cureus.29209

**Published:** 2022-09-15

**Authors:** Erdinc Cekic

**Affiliations:** 1 Otolaryngology, Haseki Training and Research Hospital, Istanbul, TUR

**Keywords:** samter’s syndrome, nasal polyps, paranasal sinus, endoscopic sinus surgery, revision

## Abstract

Background

Endoscopic sinus surgery is accepted as an effective surgical approach in the management of chronic rhinosinusitis. Different clinical pictures can be observed in chronic rhinosinusitis with nasal polyps (CRSwNP). Unfortunately, the eradication of the disease is impossible in all cases even if it is performed by experienced surgeons. In a significant number of patients, the polyps may regenerate and symptoms may reappear more or less in different durations. Due to the complex pathophysiology of the disease, revision sinus surgery is accepted as a multifactorial problem. We investigated the possible host and surgical factors which are related to increased frequency and earlier revision surgeries in CRSwNP.

Methodology

Patients operated on two or more times between 2010 and 2020 were retrospectively identified. A total of 49 patients with CRSwNP (38 male, 11 female) were statistically analyzed. The effects of host and surgical factors on the frequency and duration of revision surgery in CRSwNP were assessed.

Results

Samter’s syndrome was noted to be a significant host factor affecting recurrence and revision surgeries. In addition, we observed significantly more frequent scarring and adhesions in patients with a higher number of operations.

Conclusions

Patients with Samter’s syndrome should be informed of possible revision surgeries. The soft and mucosa-preserving technique is important for less scarring and good postoperative results.

## Introduction

Endoscopic sinus surgery is accepted as an effective and safe surgical approach in the management of chronic rhinosinusitis with or without nasal polyposis. Significant improvements in breathing problems, snoring, olfaction, and quality of life scores have been reported in the literature [1-3]. Unfortunately, eradication of the disease is impossible in all cases even when the surgery is performed by experienced surgeons using advanced technological devices. The polyps may regenerate and symptoms may come back in different durations in a significant number of patients. According to the literature, revision rates for endoscopic sinus surgery range between 4% and 19.1% [4-6]. Due to the complex pathophysiology of the disease, it is logical to accept the cause of the revision as a multifactorial problem. Asthma, Samter’s syndrome (aspirin-exacerbated respiratory disease, AERD), and cystic fibrosis are common host factors that increase the risk of revision and are accepted as factors that are almost impossible to change [5,7,8]. Interestingly, recent reports suggest that the female gender is a potential risk factor for revision surgery [5,7]. Middle turbinate lateralization, synechia, scarring, and missed natural ostium have been accepted as surgeon-dependent factors and are possible to alter [9]. Different clinical presentations can be seen in chronic rhinosinusitis with nasal polyps (CRSwNP). While polyps recur in some patients within months, in some patients, they recur after years. Therefore, some patients require several revision surgeries. In this study, we investigated the possible host and surgical factors (e.g., smoking, asthma, intraoperative findings), which are related to the recurrence of polyps and increased frequency and earlier revision surgeries in CRSwNP.

## Materials and methods

This retrospective study was approved by the local ethical committee of Health Science University, Haseki Hospital Ethical Committee, Istanbul (date: 07.05.2020, approval number: 154/2020). The study was conducted in accordance with the principles laid down in the Declaration of Helsinki. A total of 82 patients who underwent endoscopic sinus surgery two or more times between 2010 and 2020 were retrospectively identified. Hospital records, including intraoperative findings and postoperative follow-ups, were analyzed. In total, 53 of 82 patients with available records were enrolled in the study, and informed consent was obtained from all patients. Two patients had a recurrence of mucocele, one was a recurrence of an antrochoanal polyp and one was an isolated sphenoid sinus pathology. The rest of the patients had CRSPwNP (49 of 53 patients) (Table 1). To achieve a more homogenous group, only CRSwNP patients were included in the statistical assessment. Age, sex, and comorbidities of patients; the number of previous endoscopic surgeries; follow-up; and revision time (duration between the last surgery and the previous one before the last surgery) were recorded. Operation notes of patients were identified, and intraoperative findings (uncompleted anterior and posterior ethmoidectomy, frontal recess re-stenosis, sphenoid sinus re-stenosis, osteomeatal complex re-stenosis, missed natural ostium, extensive adhesions, and scarring) were recorded. Variables were divided into host and surgical factors. Sex, cigarette smoking, asthma, and Samter’s syndrome were grouped as host factors. Samter’s syndrome or AERD was defined according to the American Academy of Allergy, Asthma and Immunology as the following three medical conditions: asthma, sinus disease with recurrent nasal polyps, and sensitivity to aspirin or other non-steroidal anti-inflammatory drugs (NSAID). Intraoperative findings (uncompleted anterior and posterior ethmoidectomy, frontal recess re-stenosis, sphenoid sinus re-stenosis, osteomeatal complex re-stenosis, missed natural ostium, extensive adhesions, and scarring) and surgical navigation usage were grouped as surgical factors. The effects of host and surgical factors on the frequency and duration of revision surgery in CRSwNP were assessed.

**Table 1:** Diagnosis of patients.

Diagnosis	n	Percentage
Antrochoanal polyp	1	1.88
Mucocele	2	3.77
Isolated sphenoid sinus pathology	1	1.88
Chronic rhinosinusitis with nasal polyposis	49	92.45
Total	53	100

Statistical analysis

The software program SPSS version 15.0 for Windows (SPSS Inc., Chicago, IL, USA) was used for statistical analysis. Descriptive statistics were presented as numbers and percentages for categorical variables and as mean, standard deviation, minimum, and maximum for quantitative variables. Quantitative variables in independent groups were compared using Student’s t-test during normal distribution condition, the Mann-Whitney U test when normal distribution condition was not met, and using the Wallis test in more than two groups. Relations between quantitative variables were analyzed using Spearman correlation analysis when parametric conditions were not met. The statistical significance level was set as less than 0.05 (p < 0.05).

## Results

In this retrospective study, the most frequent cause (49/53, 92.45%) for revision surgery was nasal polyposis. A total of 49 patients with CRSwNP (38 male, 11 female) were statistically analyzed. The age of the patients ranged between 16 and 74, with a mean of 46.2 (±12.7) years. Follow-up periods ranged between six and 123 months with a mean of 46.9 (±35.8) months. Revision time is calculated as the duration (months) between the last surgery and the previous one before the last surgery. The earliest revision time was eight months, and the latest one was 276 months, with a mean revision time of 70.6 (±63.5) months. In total, 12 (12/49, 24.5%) patients were cigarette smokers of at least one package a day. Twelve (12/49, 24.5%) patients were diagnosed with asthma and received treatment before the operation, and two (2/49, 4.1%) patients were diagnosed with Samter’s syndrome (AERD).

Septoplasty was performed in three patients in previous surgeries, and in 14 patients in the last revision surgeries. A total of 17 (17/49, 34.7%) patients underwent septoplasty. Radiofrequency or cauterization for inferior turbinate was performed in four (4/49, 8.2%) patients, and partial resection of the inferior turbinate was performed only in one (1/49, 2%) patient (Table 2). The total number of endoscopic sinus surgeries of patients ranged between two and 12, with a mean of 2.7 (±1.5) operations.

**Table 2:** Some demographic findings of patients with CRSwNP. RF = radiofrequency; CRSwNP = chronic rhinosinusitis with nasal polyps

		n	Percentage
Sex	Female	11	22.4
	Male	38	77.6
Comorbidities	Asthma	12	24.5
	Samter’s syndrome	2	4.1
	Smoking	12	24.5
Additive procedures	Septoplasty	17	34.7
	RF or cauterization for inferior turbinate	4	8.2
	Partial resection for inferior turbinate	1	2

Patients were analyzed and evaluated for intraoperative findings, as presented in Table 3. Middle turbinate resection was performed in 16 (16/49, 32.7%) patients during previous surgeries. Osteomeatal complex (OMC) re-stenosis was observed in 32 (32/49, 65.3%) patients. The most frequently observed pathology was residual ethmoidal cells, uncompleted posterior ethmoidectomy was observed in 37 (37/49, 75.5%) patients, and uncompleted anterior ethmoidectomy was observed in 38 (38/49, 77.5%) patients. Frontal recess re-stenosis was observed in 33 (33/49, 67.4%) patients, and sphenoid sinus ostium re-stenosis was observed in 15 (15/49, 30.6%) patients. Extensive scarring and adhesions were observed in 10 (10/49, 20.4%) patients. Missed natural ostium of maxillary sinus recirculation phenomenon was observed only in one (1/49, 2%) patient.

**Table 3:** Intraoperative findings. OMC = osteomeatal complex

	n	Percentage
Middle turbinate resection	16	32.7
OMC stenosis	32	65.3
Uncompleted anterior ethmoidectomy	38	77.5
Uncompleted posterior ethmoidectomy	37	75.5
Frontal sinus stenosis	33	67.4
Sfenoid sinus stenosis	15	30.6
Adhesions and scarring	10	20.4
Missed natural ostium	1	2

Three operations could not be completed due to excessive bleeding. Surgical navigation was used in 12 patients, and the lacrimal sac was exposed in one patient identified using the navigation system. Any permanent or transient ocular complication was not observed postoperatively.

The effects of host factors on revision time and the number of operations were statistically analyzed (Tables 4, 5). Patients with Samter’s syndrome had a significantly higher number of operations (p = 0.006). Similarly, earlier revision surgeries were observed in patients with Samter’s syndrome; however, this difference was not statistically significant (p = 0.09). Other factors (sex, smoking, asthma) did not show any effect on revision time and number of operations.

**Table 4:** Effect of host factors on revision time. *Statistical evaluation may not be very reliable because the number of patients with Samter’s syndrome is too small.

		n (%)	Revision time (months) (mean and median)		
			Mean	Median	P-value
Sex	Female	11 (22.4)	75.4	60	0.952
	Male	38 (77.6)	69.2	52	
Smoking	Nonsmoker	37 (75.5)	70.4	49	0.408
	Smoker	12 (24.5)	70.9	57,5	
Asthma	Absence	37 (75.5)	70.4	49	0.408
	Presence	12 (24.5)	70.9	57,5	
Samter’s syndrome*	Absence	47 (95.9)	72.8	59	0.090
	Presence	2 (4.1)	18.0	18	

**Table 5:** Effect of host factors on the number of operations. *Statistical evaluation may not be very reliable because the number of patients with Samter’s syndrome is too small.

		Number of operations (mean and median)		
		Mean	Median	p
Sex	Female	3.45	2	0.353
	Male	2.42	2	
Smoking	Nonsmoker	2.65	2	0.325
	Smoker	2.67	2	
Asthma	Absence	2.65	2	0.325
	Presence	2.67	2	
Samter’s syndrome*	Absence	2.40	2	0.006
	Presence	8.50	5	

The effects of surgical factors on revision time and the number of operations were statistically (Tables 6, 7). Extensive adhesions and scarring were significantly more frequently observed in patients with a higher number of operations (p = 0.004). Other surgical factors (middle turbinate resection, OMC stenosis, uncompleted anterior and posterior ethmoidectomy, frontal re-stenosis, sphenoid re-stenosis, missed natural ostium) did not show any effect on revision time and the number of operations.

**Table 6:** Effect of surgical factors on revision time. OMC = osteomeatal complex

		n (%)	Revision time (months) (mean and median)		
			Mean	Median	P-value
Middle turbinate	Non-resected	33 (67.3)	71.7	49	0.907
	Resection	16 (32.7)	68.2	57	
Adhesions	Absence	39 (79.6)	71.9	60	0.303
	Presence	10 (20.4)	65.1	36	
OMC stenosis	Absence	17 (34.7)	57.8	48	0.360
	Presence	32 (65.3)	77.3	60	
Missed natural ostium	Absence	48 (98.0)	71.7	57	-
	Presence	1 (2.0)	14		
Frontal stenosis	Absence	16 (32.7)	54.3	54	0.694
	Presence	33 (67.4)	78.4	49	
Sphenoid stenosis	Absence	34 (69.4)	69.2	52	1.000
	Presence	15 (30.6)	73.6	59	
Uncompleted anterior ethmoidectomy	Absence	11 (22.4)	66.0	49	0.849
	Presence	38 (77.5)	67.1	55	
Uncompleted posterior ethmoidectomy	Absence	12 (24.5)	68.5	54,5	0.771
	Presence	37 (75.5)	71.2	55	
Navigation usage	No	37 (75.5)	69.6	59	0.816
	Yes	12 (24.5)	73.6	52	

**Table 7:** Effect of surgical factors on the number of operations. OMC = osteomeatal complex

		Number of operations (mean and median)		
		Mean	Median	P-value
Middle turbinate	Nonresected	2.73	2	1.000
	Resection	2.50	2	
Adhesions	Absence	2.56	2	0.004
	Presence	3.00	2.75	
OMC stenosis	Absence	2.47	2	0.824
	Presence	2.75	2	
Missed natural ostium	Absence	2.67	2	-
	Presence	2		
Frontal stenosis	Absence	3.06	2	0.967
	Presence	2.52	2	
Sphenoid stenosis	Absence	2.38	2	0.207
	Presence	3.27	2	
Uncompleted anterior ethmoidectomy	Absence	2.27	2	0.283
	Presence	2.83	2	
Uncompleted posterior ethmoidectomy	Absence	2.25	2	0.268
	Presence	2.78	2	
Navigation usage	No	2.73	2	0.774
	Yes	2.42	2	

## Discussion

Management of CRSwNP includes different medical and surgical treatment modalities but has limited success in eliminating the disease. In addition to the well-known traditional therapeutics such as systemic or topical corticosteroids, novel therapeutics targeting type 2 inflammation are now coming to the front. Omalizumab (anti-IgE antibody), mepolizumab (anti-IL-5 antibody), and dupilumab (anti-IL-4 ve IL-13 antibody) are some important examples of these new drugs [10-12]. High-definition, different-angle endoscopes, endovision systems, and integrated surgical navigation systems are important technological advancements in treating paranasal sinus diseases. Additionally, because modern therapeutics and advanced technological devices cannot eradicate polyps entirely, chronic drug usage and recurrent surgeries may become inevitable in some patients with CRSwNP. Our aim in endoscopic sinus surgery is the restoration of normal sinus physiology. To solve the problems of patients, it is essential to identify the source of the persistent disease. According to previous studies, predictive factors affecting polyp recurrence include asthma, aspirin sensitivity, and previous endoscopic sinus surgery history. These factors have been considered in relation to the early and higher number of revision surgeries [7,8,13-15].

Mendelsohn et al. [15] categorized patients with nasal polyposis as Samter’s syndrome, asthma, and control groups in their study. They investigated polyp recurrence and revision surgery necessity in each group. Polyp recurrence and the risk of early revision surgery were found to be significantly higher in patients with Samter’s syndrome. Similarly, in our study, patients with Samter’s syndrome had an earlier and higher number of surgeries. Note that statistical evaluation for Samter’s syndrome may not be very reliable as the number of patients with Samter’s syndrome was too small. In the statistical evaluation, only the number of operations was significant. Despite the intense medical treatment such as inhalers and nasal corticosteroids, patients with Samter’s syndrome usually need multiple revision surgeries for polyp recurrence. Aspirin desensitization and aggressive steroid douching to postoperative cavities may be good alternative treatments to decrease the regeneration of polyps and the revision risk of patients [16].

In our study, asthma was not a significant factor in revision time and frequency of recurrent surgeries. The severity and frequency of asthma attacks were not known; hence, the proper assessment of the effect of asthma on revision surgeries could not be achieved correctly. The small number of patients with asthma for proper analysis is a limitation of our study. Wu et al. [17] found smoking to be a significant factor in decreasing the revision time and associated this finding with the negative effect of smoking on the nasal mucosa. In our study, we did not find any difference in revision time and the number of operations on smoker or non-smoker patients. It is very difficult to demonstrate the negative effects of smoking on the nasal mucosa without a histopathological examination.

As we evaluated the surgical factors, the most frequently observed intraoperative finding in our study was uncompleted anterior or posterior ethmoidectomies and residual ethmoidal cells. We associate this finding with our mucosa-preserving, limited surgical approach and the complex anatomy of ethmoidal cells rather than the wide cavity approach. Muse and Kauntakis [18], in their study, reported the most frequent intraoperative findings as lateralized middle turbinate, uncompleted anterior ethmoidectomy, and frontal recess stenosis. Additionally, Ramadan [19], in his study, reported the intraoperative findings in patients with revision surgeries due to surgical failures as residual cells in the ethmoid and frontal recess region. Interestingly, he also reported the missed natural ostium of the maxillary sinus and the recirculation phenomenon in approximately 16% of patients. In our study, we observed this phenomenon in only one patient.

As we statistically analyzed these intraoperative findings according to the effect on revision time and the number of recurrent surgeries, only extensive adhesions and scarring were significantly observed in patients with a higher number of surgeries (p = 0.004). We thought that recurrent surgery results in more adhesions and scarring in postsurgical cavities. Chambers et al. [20] found that scarring in the middle meatus and ethmoid region causes unsatisfactory results in patients. Poor and aggressive surgical techniques may result in excessive scarring, which may cause synechia formation, lateralization of the middle turbinate, circumferential scarring, and re-stenosis of the ostia [21]. Consequently, scarring leads to a vicious circle: more scarring causes more surgeries, and more surgeries cause more scarring. We think that a soft and mucosa-preserving technique is important for good postoperative results.

Interestingly, Wu et al. [17] found a significant delay in revision surgery in patients with middle turbinate resection. They associated this finding with increased space after turbinectomy and the expansion of topical steroids easier. However, they warn surgeons to pay extra attention to middle turbinate resection due to increased surgical risk, e.g., cerebrospinal fluid fistula. Unlike this study, we did not find any difference in both revision time and the number of recurrent surgeries in resected middle turbinate and non-resected patients. According to our principles, we advocate preserving the middle turbinate and its mucosa as much as possible to achieve normal nasal flow and prevent adhesion formation due to lateralized middle turbinate and important surgical landmarks for subsequent surgeries.

In this study, factors related to revision endoscopic surgery were categorized as host and surgical factors. We found Samter’s syndrome to be a significant host factor for recurrence and revision surgeries. In addition, we observed significantly more frequent scarring and adhesions in patients with a higher number of operations.

Study limitations

Because of the subjective evaluation and absence of standardization for intraoperative findings, it is difficult to evaluate and compare the studies related to revision endoscopic sinus surgeries without bias. The limitations of our study are the retrospective design and lack of standardization of surgery. It would be difficult to achieve a prospective design and single surgeon approach. In addition, patients’ compatibility with postoperative treatment was not measured in this study.

## Conclusions

Patients with Samter’s syndrome should be informed of possible revision surgeries. Aspirin desensitization and intense postoperative treatments may be used to lower the risk of recurrent and earlier surgeries. The soft and mucosa-preserving technique is important for less scaring and good postoperative results.
